# Joint Commission Primary Stroke Centers Utilize More rt‐PA in the Nationwide Inpatient Sample

**DOI:** 10.1161/JAHA.112.000071

**Published:** 2013-04-24

**Authors:** Michael T. Mullen, Scott E. Kasner, Michael J. Kallan, Dawn O. Kleindorfer, Karen C. Albright, Brendan G. Carr

**Affiliations:** 1 Department of Neurology, University of Pennsylvania, Philadelphia, PA (M.T.M., S.E.K.); 2 Department of Emergency Medicine, University of Pennsylvania, Philadelphia, PA (B.G.C.); 3 Center for Clinical Epidemiology & Biostatistics, University of Pennsylvania, Philadelphia, PA (M.J.K., B.G.C.); 4 Department of Neurology, University of Cincinnati, Cincinnati, OH (D.O.K.); 5 Health Services and Outcomes Research Center for Outcome and Effectiveness Research and Education, University of Alabama at Birmingham, Birmingham, AL (K.C.A.); 6 Center of Excellence in Comparative Effectiveness Research for Eliminating Disparities/Minority Health & Health Disparities Research Center, University of Alabama at Birmingham, Birmingham, AL (K.C.A.); 7 Department of Epidemiology, University of Alabama at Birmingham, Birmingham, AL (K.C.A.); 8 Department of Neurology, University of Alabama at Birmingham, Birmingham, AL (K.C.A.)

**Keywords:** acute stroke, cerebral infarction, outcomes research, systems of care, thrombolysis

## Abstract

**Background:**

The Joint Commission began certifying primary stroke centers (PSCs) in December 2003 and provides a standardized definition of stroke center care. It is unknown if PSCs outperform noncertified hospitals. We hypothesized that PSCs would use more recombinant tissue plasminogen activator (rt‐PA) for ischemic stroke than would non‐PSCs.

**Methods and Results:**

Data were obtained from the Nationwide Inpatient Sample from 2004 to 2009. The analysis was limited to states that publicly reported hospital identity. All patients ≥18 years with a primary diagnosis of acute ischemic stroke were included. Subjects were excluded if the treating hospital was not identified, if it was not possible to determine the temporal relationship between certification and admission, and/or if admitted as a transfer. Rt‐PA was defined by ICD9 procedure code 99.10. All eligibility criteria were met by 323 228 discharges from 26 states. There were 63 145 (19.5%) at certified PSCs. Intravenous rt‐PA was administered to 3.1% overall: 2.2% at non‐PSCs and 6.7% at PSCs. Between 2004 and 2009, rt‐PA administration increased from 1.4% to 3.3% at non‐PSCs and from 6.0% to 7.6% at PSCs. In a multivariable model incorporating year, age, sex, race, insurance, income, comorbidities, DRG‐based disease severity, and hospital characteristics, evaluation at a PSC was significantly associated with rt‐PA utilization (OR, 1.87; 95% CI, 1.61 to 2.16).

**Conclusions:**

Subjects evaluated at PSCs were more likely to receive rt‐PA than those evaluated at non‐PSCs. This association was significant after adjustment for patient and hospital‐level variables. Systems of care are necessary to ensure stroke patients have rapid access to PSCs throughout the United States.

## Introduction

Treatment with recombinant tissue plasminogen activator (rt‐PA) has been shown to improve outcome after acute ischemic stroke. Unfortunately, only a small percentage of stroke patients receive this important therapy, with estimates ranging from 2.4% to 9%.^1–3^ Furthermore, studies suggest that fewer than half of patients who are eligible for rt‐PA actually receive treatment.^4–8^

Healthy People 2020, a report of the US Department of Health and Human Services that highlights the nation's 10‐year goals for health promotion and disease prevention, includes a 20% reduction in stroke mortality and an increase in thrombolytic therapy for acute stroke among the nation's public health priorities.^9^ Creating a system of care for acute stroke that ensures rapid access to specialized stroke centers across the United States is critical to achieving these goals.^10^ Primary stroke centers (PSCs) should function as the basic building blocks of this system, which means that they must be prepared to administer acute stroke therapies.^11–12^

Although there are competing definitions of a PSC, The Joint Commission (TJC) provides a standardized, nationwide definition of stroke center care. The PSC certification process is based on criteria proposed by the Brain Attack Coalition, a group of 17 national organizations dedicated to improving outcomes in stroke.^11,13^ These criteria have been shown to correlate with increased utilization of acute stroke therapies at selected academic medical centers; however, data comparing TJC PCSs to non‐PSCs are limited.^14^ Studies have shown a modest reduction in mortality at PSCs, but differences may be attributable to baseline differences in hospital performance rather than certification.^15–17^ Studies in Medicare recipients and in the state of Illinois have shown increased rt‐PA utilization at certified hospitals in those populations.^18–19^ We aimed to compare utilization of rt‐PA at PSCs and non‐certified hospitals in a nationwide all‐payer, age≥18 cohort. We hypothesized that PSCs would administer rt‐PA to a significantly higher proportion of patients than would non‐PSCs.

## Methods

### Study Population

For this retrospective cohort study, data were obtained from the Nationwide Inpatient Sample (NIS), Healthcare Cost and Utilization Project (HCUP), Agency for Healthcare Research and Quality for full calendar years 2004–2009. The NIS is the largest all‐payer inpatient database in the United States. It is designed to represent a 20% stratified sample of US hospitals. The NIS contained data from 37 states in 2004, of which 25 publicly reported hospital identity. This increased to 44 states in 2009, of which 26 identified hospitals (Table S1). Additional details on the Nationwide Inpatient Sample can be obtained from the Healthcare Cost and Utilization Project.^20^

The analysis was limited to discharged patients with a principal diagnosis of ischemic stroke (ICD9 codes 433.x1, 434.x1, 436), age ≥18 years, and admitted in a state that publicly reports hospital identity. The selected ICD9 codes have a positive predictive value exceeding 85% and are recommended by the US Food and Drug Administration Mini‐Sentinel project to identify acute ischemic stroke (AIS) in administrative data sets.^21^ Subjects who were admitted as a transfer from another acute care facility were excluded as they were not likely to arrive within rt‐PA treatment windows. Subjects who were missing sex, length of stay, or primary payer were also excluded (0.2% of sample). Included subjects had complete data for all variables except for race, which is not reported by all states in the NIS. The date of initial certification for PSCs was obtained from TJC on May 17, 2011. For each patient it was determined if the treating hospital was certified at the time of admission. In a small subset of subjects (0.4%) it could not be determined if the treating hospital was a certified PSC at the time of admission, and these subjects were excluded from the analysis.

### Covariates

The primary outcome was treatment with intravenous (IV) rt‐PA. IV rt‐PA was defined as an ICD9 procedure code of 99.10. Patient‐level variables included year of discharge, age, sex, race, primary expected payer (Medicare, Medicaid, private including health maintenance organization (HMO), self‐pay, no charge, other), and median household income, by quartile, in the patient's ZIP code. Multivariable models also incorporated 29 of the Elixhauser medical comorbidities (Table S2) and an all‐patient refined diagnosis‐related group (APR‐DRG) based mortality risk indicator.^22–23^ The APR‐DRG mortality risk system incorporates diagnoses, procedures, and age to assign each subject a score from 1 to 4, indicating minor, moderate, major, or extreme risk of mortality.

Hospital‐level variables included teaching status (yes/no), location (urban/rural), hospital region (East, Midwest, South, West), and acute ischemic stroke volume (<100, 100 to 299, 300+ discharges/year).

### Statistical Analysis

Baseline characteristics for patients treated at PSCs and those treated at noncertified centers were described using measures of central tendency (means, medians) for continuous variables and proportions for categorical variables. Group differences were evaluated using the Student's *t*, Wilcoxon rank‐sum, and *χ*^2^ tests as appropriate. The proportion of subjects receiving rt‐PA was calculated for each year of the analysis for both PSCs and non‐PSCs. Univariate and multivariable logistic regression were used to assess the relationship between PSC certification and rt‐PA administration. The Nationwide Inpatient Sample uses a complex survey design that includes stratification, clustering, replication, and unequal probabilities of selection into the design. Our analysis was further complicated because we limited it to a specific subpopulation (patients with stroke who were admitted in states that report hospital identity), creating a convenience sample. Our analytic models used survey statistics and Taylor series estimation to account for the survey design and clustering within hospitals. The procedures used were consistent with recommendations outlined in the Healthcare Cost and Utilization Project data documentation (http://www.hcup-us.ahrq.gov/reports/methods/CalculatingNISVariances200106092005.pdf). Three multivariable models were constructed. The first model incorporated year of discharge, age, sex, race, primary expected payer, median income by zip code, hospital region, teaching status, location, and ischemic stroke admission volume. The second model incorporated all variables from the first model plus 29 of the Elixhauser comorbid conditions. The third model incorporated all variables from the second model plus APR‐DRG risk of mortality. Rt‐PA utilization was compared at PSCs and non‐PSCs in predefined subgroups of age strata (<55, 55 to 64, 65 to 74, 75 to 84, 85+ years), hospital teaching status, hospital location, and volume of acute stroke admissions. The number of hospitals and the range of cases per year within each of these subgroups are presented in Table S3. Finally, in a post hoc analysis we evaluated for an interaction between ischemic stroke admission volume and PSC certification using the fully adjusted model. Analysis was conducted in SAS‐callable‐SUDAAN version 10.0.1.

## Results

**Figure 1.**
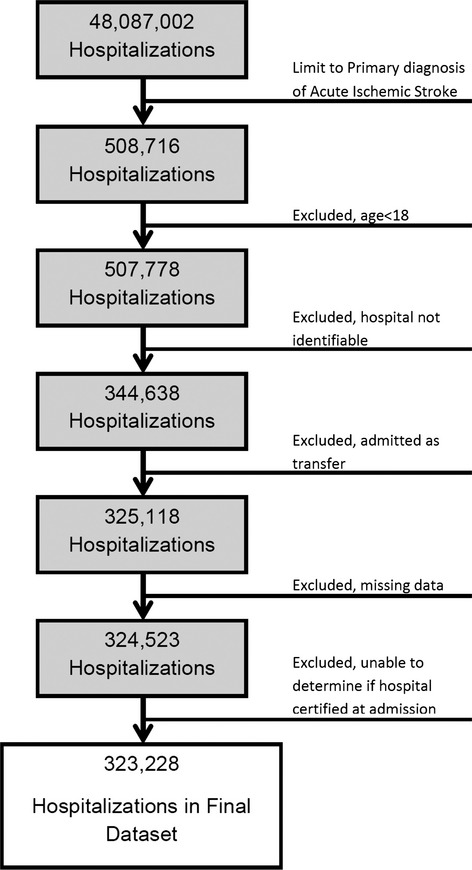
There were 48 087 002 subjects in the data set, of whom 508 716 had a primary diagnosis of acute ischemic stroke. After all exclusion criteria, the final study population was 323 228.

From 2004 to 2009 there were 508 716 subjects with a primary diagnosis of ischemic stroke. Of these, 323 228 met all inclusion criteria (Figure 1). The most common reason for exclusion was admission in a state that does not publicly disclose hospital identity. The demographics of subjects treated at PSCs and non‐PSCs are summarized in Table 1.

**Table 1.** Subject and Hospital Characteristics

	Total Population	PSC	Non‐PSC
Number of subjects	323 228	63 145	260 083
Age, y (mean)	72.2	71.3	72.4
Female	53.8%	52.2%	54.1%
Race/ethnicity*			
White	56.4%	56.0%	56.5%
Black	11.5%	12.8%	11.1%
Asian/Pacific Islander	2.4%	3.2%	2.2%
Native American	0.3%	0.2%	0.3%
Hispanic	6.3%	3.9%	6.9%
Other	1.8%	2.5%	1.6%
Missing	21.4%	21.3%	21.4%
Payment type			
Medicare	68.0%	64.3%	68.9%
Medicaid	6.4%	6.0%	6.4%
Private, including HMO	19.5%	23.2%	18.6%
Self‐pay	3.6%	3.8%	3.5%
No charge	0.5%	0.5%	0.5%
Other	2.1%	2.2%	2.1%
Median household income, by ZIP code			
Lowest quartile	24.3%	20.5%	25.2%
Second quartile	25.4%	22.6%	26.1%
Third quartile	23.9%	23.7%	23.9%
Highest quartile	24.1%	31.3%	22.4%
Missing	2.2%	1.8%	2.3%
Teaching hospital	40.2%	61.5%	35.1%
Hospital location (rural)	13.1%	1.6%	15.8%
Ischemic stroke volume (cases/year)			
<100	16.6%	1.4%	20.3%
100 to 299	46.2%	30.1%	50.1%
300+	37.2%	68.5%	29.6%

PSC vs non‐certified hospitals: Student *t* test for age, *χ*^2^ test for all other variables. PSC indicates primary stroke center; HMO, health maintenance organization; NIS, Nationwide Inpatient Sample.

* Race and ethnicity not reported separately in the NIS.

**Figure 2.**
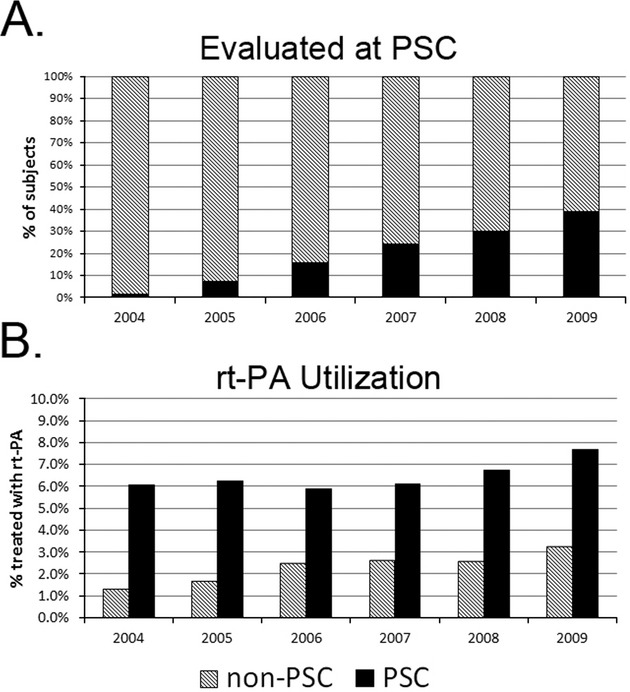
A, The percentage of patients evaluated at a primary stroke center increased from 1.4% in 2004 to 39.5% in 2009. B, The percentage of patients treated with rt‐PA at non‐PSCs was 1.4% in 2004, increasing to 3.3% in 2009. The percentage of patients treated with rt‐PA at PSCs was 6.0% in 2004, increasing to 7.6% in 2009. PSC indicates primary stroke center; rt‐PA, recombinant tissue plasminogen activator.

In total, 63 145 patients (19.5%) were evaluated at hospitals that were TJC‐certified PSCs at the time of admission and 260 083 (80.5%) at non‐PSCs. The number of PSCs increased from 112 on December 31, 2004, to 673 on December 31, 2009. In 2004, 745 of 55 176 patients (1.4%) were treated at a certified PSC, whereas in 2009, 20 322 of 51 421 patients (39.5%) were treated at a PSC (Figure 2A). The proportion of patients receiving rt‐PA was 3.1% overall: 2.2% at non‐PSCs and 6.7% at PSCs. Between 2004 and 2009 the annual percentage of rt‐PA administration increased from 1.4% to 3.3% at non‐PSCs and 6.0% to 7.6% at PSCs (Figure 2B). In univariate analysis certification was associated with rt‐PA treatment (OR, 3.14; 95% CI, 2.74 to 3.60). In all 3 multivariable models PSC certification was significantly associated with rt‐PA treatment. The results were similar across all 3 models: model 1, which incorporated demographics and hospital characteristics (OR, 1.89; 95% CI, 1.65 to 2.17); model 2, which added Elixhauser comorbidities (OR, 1.87; 95% CI, 1.62 to 2.17; and model 3, which added APR‐DRG mortality risk (OR, 1.87; 95% CI, 1.61 to 2.16). The association of certification and rt‐PA utilization was significant in all years, but the effect size was strongest in 2004 (OR, 2.95; 95% CI, 1.74 to 5.00; model 3) and weakest in 2009 (OR, 1.67; 95% CI, 1.26 to 2.20; model 3). Results for all models in each year are reported in Table 2. The odds of rt‐PA treatment were higher at PSCs than at non‐PSCs in all subgroups, as shown in Table 3. The interaction between ischemic stroke admission volume and certification was not statistically significant (*P*=0.29).

**Table 2.** Odds of rt‐PA Treatment, PSC Versus Non‐PSC

	Unadjusted OR (95% CI)	Model 1	Model 2	Model 3
		Adjusted OR (95% CI)*	Adjusted OR (95% CI)*	Adjusted OR (95% CI)*
All Years	3.14 (2.74 to 3.60)	1.89 (1.65 to 2.17)	1.87 (1.62 to 2.17)	1.87 (1.61 to 2.16)
2004 only	4.68 (3.07 to 7.13)	2.91 (1.69 to 4.99)	2.96 (1.74 to 5.02)	2.95 (1.74 to 5.00)
2005 only	3.78 (2.45 to 5.84)	2.91 (1.90 to 4.45)	2.82 (1.73 to 4.59)	2.82 (1.74 to 4.56)
2006 only	2.41 (1.82 to 3.19)	1.84 (1.43 to 2.38)	1.79 (1.35 to 2.37)	1.77 (1.33 to 2.34)
2007 only	2.40 (1.91 to 3.01)	1.80 (1.46 to 2.23)	1.94 (1.59 to 2.36)	1.93 (1.58 to 2.35)
2008 only	2.68 (2.15 to 3.35)	1.95 (1.58 to 2.39)	1.92 (1.53 to 2.41)	1.92 (1.53 to 2.41)
2009 only	2.45 (1.93 to 3.11)	1.70 (1.35 to 2.14)	1.67 (1.26 to 2.20)	1.67 (1.26 to 2.20)

rt‐PA indicates recombinant tissue plasminogen activator; PSC, primary stroke center; OR, odds ratio; CI, confidence interval; AHRQ, Agency for Healthcare Research and Quality; APR‐DRG, all‐patient refined diagnosis‐related group.

* Model 1 adjusted for year, age, sex, race, primary payer, median income quartiles by ZIP, hospital region, teaching status of hospital, hospital location, volume of AIS.

* Adjusted for all in model 1 plus each of the 29 AHRQ (individual) comorbidities.

* Adjusted for all in model 2 plus APR‐DRG risk mortality (minor, moderate, major, and extreme likelihood of dying).

**Table 3.** Odds of rt‐PA Treatment, PSC Versus Non‐PSC, Stratified Analysis

	Unadjusted OR (95% CI)	Model 1	Model 2	Model 3
		Adjusted OR (95% CI)*	Adjusted OR (95% CI)*	Adjusted OR (95% CI)*
Patient age (y)				
<55	2.50 (2.11 to 2.96)	1.71 (1.40 to 2.08)	1.62 (1.31 to 2.01)	1.62 (1.31 to 2.00)
55 to 64	2.80 (2.38 to 3.30)	1.80 (1.51 to 2.14)	1.79 (1.49 to 2.16)	1.77 (1.47 to 2.13)
65 to 74	2.77 (2.37 to 3.22)	1.77 (1.50 to 2.07)	1.81 (1.51 to 2.17)	1.80 (1.50 to 2.17)
75 to 84	3.59 (3.09 to 4.16)	2.05 (1.76 to 2.39)	2.00 (1.69 to 2.37)	1.99 (1.68 to 2.35)
85+	4.00 (3.32 to 4.83)	2.12 (1.75 to 2.58)	2.21 (1.78 to 2.76)	2.20 (1.76 to 2.75)
Hospital‐level variables				
Teaching status				
Teaching hospital	2.51 (2.12 to 2.98)	1.84 (1.53 to 2.20)	1.79 (1.49 to 2.15)	1.78 (1.48 to 2.14)
Nonteaching hospital	3.21 (2.59 to 3.96)	2.01 (1.65 to 2.44)	2.10 (1.67 to 2.66)	2.10 (1.67 to 2.65)
Location				
Urban	2.85 (2.49 to 3.27)	1.88 (1.64 to 2.16)	1.86 (1.60 to 2.16)	1.85 (1.59 to 2.15)
Rural	3.11 (1.50 to 6.46)	3.52 (1.61 to 7.66)	4.67 (1.80 to 12.14)	4.58 (1.78 to 11.76)
Ischemic stroke volume (cases/year)				
<100	2.49 (1.67 to 3.71)	2.23 (1.46 to 3.38)	1.87 (1.08 to 3.22)	1.89 (1.10 to 3.24)
100 to 299	2.77 (2.36 to 3.26)	2.20 (1.88 to 2.58)	2.15 (1.83 to 2.52)	2.15 (1.83 to 2.52)
300+	2.25 (1.85 to 2.72)	1.76 (1.45 to 2.14)	1.77 (1.43 to 2.20)	1.76 (1.42 to 2.19)

rt‐PA indicates recombinant tissue plasminogen activator; PSC, primary stroke center; OR, odds ratio; CI, confidence interval; AHRQ, Agency for Healthcare Research and Quality; APR‐DRG, all‐patient refined diagnosis‐related group.

* Model 1 adjusted for year, age, sex, race, primary payer, median income quartiles by ZIP, hospital region, teaching status of hospital, hospital location, and volume of AIS.

* Adjusted for all in model 1 plus each of the 29 AHRQ (individual) comorbidities.

* Adjusted for all in model 2 plus APR‐DRG risk mortality (minor, moderate, major, and extreme likelihood of dying).

## Discussion

PSCs treated a higher proportion of patients with rt‐PA than did non‐PSCs in this all‐payer cohort of adults (age≥18). Certification was associated with increased rt‐PA utilization in all age and hospital strata. These results add to the mounting evidence that TJC‐certified PSCs outperform noncertified hospitals with respect to rt‐PA use.^18–19^ Our results support the use of TJC PSCs as the building blocks of the US acute stroke care system.

In our analysis, the proportion of patients treated with rt‐PA at PSCs was relatively stable from 2004 to 2009; in comparison, the proportion of patients treated with rt‐PA at non‐PSCs more than doubled. It is not known why this occurred. Expansion of the rt‐PA treatment window to 4.5 hours after publication of ECASS‐III in 2008 may have contributed to this phenomenon.^24^ Hospitals without certification may be preparing for certification, participating in quality improvement initiatives, such as Get with the Guidelines, or be using telestroke programs, all of which may lead to increased rt‐PA utilization.

The association of certification with increased rt‐PA use was relatively stable across age strata and hospital variables. The association between PSC certification and rt‐PA use was stronger in rural centers than in other subgroups. However, there were a small number of hospitals and discharges in this subgroup, so these results should be interpreted with caution. Further work is needed to determine whether certification is equally effective in all hospital types and to ensure that all PSCs are functioning at a high level.

This study has several important limitations. The analysis was limited to identifiable hospitals in 24 to 26 states, depending on year. Although this diverse sample should provide excellent generalizability, the results are not truly nationally representative and should be regarded as a large convenience sample of US hospitals. TJC certification is not the only PSC certification in the United States. We were unable to account for state‐based or other certified PSCs. This misclassification may have biased the results toward the null. ICD9‐based definitions of rt‐PA are imprecise and known to underestimate rt‐PA utilization.^2,25^ In a prior study that compared the ICD9 procedure code of 99.10 to pharmacy billing records, ICD9 codes identified 77% of IV rt‐PA cases.^2^ As a result, the percentages reported in this article are likely an underestimate. Nonetheless, comparisons of relative rt‐PA utilization across hospitals are still informative. The odds ratios presented here would only be biased if there was differential misclassification of rt‐PA use between PSCs and non‐PSCS. Financial incentives for rt‐PA, which began in late 2005, apply equally to all hospitals and should reduce variability in rt‐PA coding. The use of administrative data precludes an evaluation of rt‐PA eligibility. Differences in eligibility across hospitals could have biased our findings; unfortunately, there is no way to test for this bias in the current study. Despite these limitations, our study contributes significantly to the literature. Reports from registries or quality improvement initiatives have more detailed clinical information, but data are only included from a select group of hospitals. Our study provides data on clinical practice across a wide sample of US hospitals, including those that choose not to participate in registries, thus providing important insights into the US healthcare system.

In conclusion, TJC provides a standardized, national definition of a primary stroke center. Patients treated at a TJC‐certified PSC are more likely to receive rt‐PA than those treated at noncertified hospitals. Further work is needed to determine whether certification is equally effective in all hospital types and to ensure that all PSCs are functioning at a high level. Systems of care are necessary to ensure that stroke patients have timely access to PSCs.
